# Adaptive designs based on the truncated product method

**DOI:** 10.1186/1471-2288-5-30

**Published:** 2005-09-19

**Authors:** Markus Neuhäuser, Frank Bretz

**Affiliations:** 1 Institute for Medical Informatics, Biometry and Epidemiology, University of Duisburg-Essen, Hufelandstr. 55, D-45122 Essen, Germany; 2 Novartis Pharma AG, WSJ-27.1.005, 4002 Basel, Switzerland

## Abstract

**Background:**

Adaptive designs are becoming increasingly important in clinical research. One approach subdivides the study into several (two or more) stages and combines the *p*-values of the different stages using Fisher's combination test.

**Methods:**

Alternatively to Fisher's test, the recently proposed truncated product method (TPM) can be applied to combine the *p*-values. The TPM uses the product of only those *p*-values that do not exceed some fixed cut-off value. Here, these two competing analyses are compared.

**Results:**

When an early termination due to insufficient effects is not appropriate, such as in dose-response analyses, the probability to stop the trial early with the rejection of the null hypothesis is increased when the TPM is applied. Therefore, the expected total sample size is decreased. This decrease in the sample size is not connected with a loss in power. The TPM turns out to be less advantageous, when an early termination of the study due to insufficient effects is possible. This is due to a decrease of the probability to stop the trial early.

**Conclusion:**

It is recommended to apply the TPM rather than Fisher's combination test whenever an early termination due to insufficient effects is not suitable within the adaptive design.

## Background

Randomized controlled experiments were introduced by Sir Ronald A. Fisher in the 1920s for agricultural studies and not in order to compare the effects of different treatments in humans. However, according to Palmer [1] the way clinical trials are conducted today is essentially unchanged from Fisher's day. In contrast to agricultural studies most clinical trials require periodic monitoring of the accumulating data, e.g. to minimize the number of experimental patients who will continue with an inferior treatment [[2], p. 360].

Adaptive designs with at least one interim analysis can potentially be used for periodic monitoring. All information from the first stage(s) can be used to plan the following stage(s). A number of adaptive designs have been proposed recently, for an overview see Bauer et al. [3]. Here, we consider the adaptive procedure according to Bauer and Köhne [4] that uses Fisher's product test.

Let *k *be the number of stages (i.e., there are *k *- 1 interim analyses), and let *p*_*i *_be the one-sided *p*-value observed with the *i*-th stage's data, *i *= 1, ..., *k*. According to Fisher's product criterion [[5], pp. 37–39] the null hypothesis H_0 _can be rejected at the end of the trial if

,

where  is the (1 - α)-quantile of the central χ^2^-distribution with 2*k *degrees of freedom.

In clinical trials boundaries for early stopping after an interim analysis may be incorporated. Obviously, in the case of *p*_1 _≤ *c*_α _early stopping with the rejection of H_0 _is possible after stage one. In general, H_0 _can be rejected after the *j*-th stage if . In addition, one may terminate the trial due to insufficient effects. A lower limit α_0 _can be included so that the trial is terminated without rejecting H_0 _if *p*_1 _≥ α_0_. According to Bauer and Köhne [[4], p. 1031] a value of 0.5 may be a suitable choice for α_0_. Bauer and Röhmel [[6], p. 1596] recommended α_0 _= 1 for establishing a dose-response relationship, that is, no early stopping without rejecting H_0 _at all. In this context, an early stopping due to insufficient effects is not feasible since doses in a plateau region could have been used. In that case, different doses may be used in the following stage.

Note that, in case of α_0 _< 1, larger boundaries for  apply for early stopping with the rejection of H_0_. For a two-stage design, one can reject H_0 _after stage one if *p*_1 _≤ α_1 _for a value of α_1 _that lies between *c*_α _and α [4,6]. This value can be calculated iteratively using the formula [[4], p. 1032]



**Figure 1:**
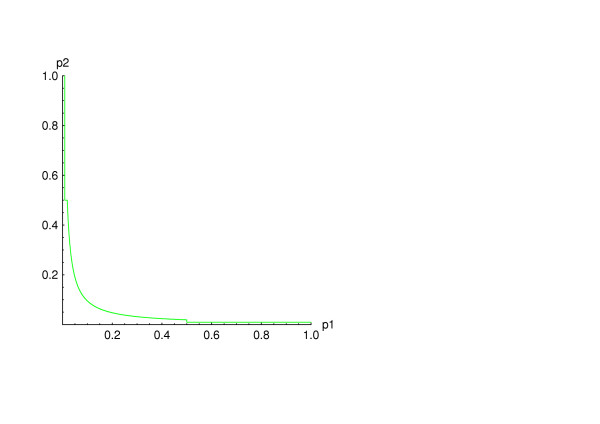
The rejection region of the truncated product method for *k *= 2 and τ = 0.5.

As an alternative to Fisher's product test, Zaykin et al. [7] recently introduced a truncated product method for combining *p*-values. To be precise, instead of calculating the product of all *p*-values, they suggested the use of the product of only those *p*-values that do not exceed some fixed cut-off value τ, 0 < τ ≤ 1. The truncated product *W*_τ _is defined as



where *I*(.) is the indicator function. Since the *p*-values of the different stages are independent,



[[7], p. 173] holds for *w *< 1 under the overall null hypothesis (i.e., under the assumption that each stage's null hypothesis is true). Figure 1 displays the rejection region for *k *= 2 and τ = 0.5.

When using the truncated product method, the (1-α)-quantile of the distribution of *W*_τ_, , is the critical value for the combination test. Analogous to Fisher's combination test an  can be calculated for given α_0 _such that the overall type I error rate is α.

Zaykin et al. [7] and Neuhäuser [8] investigated the truncated product method for combining a large number of *p*-values and demonstrated by simulation that it can provide high power. In this paper we investigate whether the truncated product method is also useful for the adaptive design described above. In contrast to previous applications [7,8] we consider classical experimental questions involving only few *p*-values. Very recently, a rank truncated product was proposed as a further alternative [9]. That method uses the product of the *K *most significant *p*-values where *K *can be chosen. Since we consider the combination of 2 to 4 *p*-values only, the rank truncated product does not seem to be appropriate for our aim.

We first present the comparison of the combinations with and without truncation for designs with two stages. Afterwards, designs with more than two stages are investigated. We then illustrate the method using two examples, and conclusions are given in final section.

## Methods

In order to compare the adaptive procedures with and without truncation we consider the situation of two parallel groups with means μ_1 _and μ_2_. There are 100 observations per stage. These observations are subdivided into two groups and are assumed to be normally distributed with a common, but unknown variance σ^2^. Student's *t *test is performed in each of the two stages with a one-sided significance level of α = 5%.

The overall *p*-value, i.e. the *p*-value of the combination test, is defined as follows [10]: In case the study stops after stage 1, the overall *p*-value equals *p*_1_. Otherwise, the overall p-value is  for Fisher's combination test and  for the truncated product test.

### The case α_0 _= 0.5

First, we consider a study that is terminated early due to insufficient effects if *p*_1 _≥ α_0 _= 0.5. Without any truncation (i.e., τ = 1) we have *c*_α _= 0.0087 and α_1 _= 0.0233 in this case [4]. However, when we set τ = α_0 _= 0.5, a smaller value for α_1 _but a larger boundary for  is obtained. To be precise, the trial can be terminated early with the rejection of H_0 _if *p*_1 _≤  = 0.0190, and there is a significance at the end of the trial if *W*_τ = 0.5 _≤  = 0.0095.

Although α_1 _is decreased the overall power can increase in case of truncation as the boundary  for *W*_τ = 0.5 _is larger than that for *W*_τ = 1_. Table I displays the overall power, that is, the power to reject H_0 _after any stage, for different alternatives  (see the appendix for details about the calculation of the power). The power is slightly higher in case of truncation. The difference is very small when the ratio (sample size in stage one)/(sample size in stage two) is large. The reason is that the probability to stop already after the first stage depends on the sample size in stage one.

**Table 1:** Power to reject H_0 _in a two-stage design with α_0_ = 0.5 (combination of *t *tests, one-sided, α = 0.05)

δ =	0.1	0.2	0.3	0.4	0.5
25 observations per group in stage one, 75 observations per group in stage two					
τ = 1	0.149	0.343	0.595	0.808	0.929
τ = 0.5	0.153	0.352	0.605	0.815	0.931

50 observations per group and stage					
τ = 1	0.162	0.377	0.644	0.854	0.959
τ = 0.5	0.165	0.384	0.652	0.860	0.961

75 observations per group in stage one, 25 observations per group in stage two					
τ = 1	0.166	0.386	0.654	0.860	0.962
τ = 0.5	0.167	0.389	0.657	0.863	0.963

The area of the rejection region of Fisher's test that can be relocated in case of α_0 _< 1 [[4], p.1032] has, under H_0_, the probability Pr(*p*_1 _≥ α_0 _and *p*_1_*p*_2 _≤ *c*_α_) = *c*_α_(-lnα_0_). In case of truncation with τ = α_0_, an area with probability Pr(*p*_1 _≥ α_0 _and *p*_2 _≤ ) =  (1 - τ) can be relocated. Since *c*_α_(-lnα_0_) >  (1 - τ) for practically relevant situations (see e.g. Table II), we have  < α_1_. Hence, the probability to terminate the trial after stage one is lower in case of truncation with τ = α_0_.

**Table 2:** Boundaries *c*_α _and  for two to four stages

Number of stages (*k*)	*c*_α_	for τ = 0.5
α = 0.025		
2	0.00380	0.00408
3	0.00072	0.00085
4	0.00015	0.00020

α = 0.05		
2	0.00870	0.00948
3	0.00184	0.00222
4	0.00042	0.00057

For instance, in the case of 50 observations per group and stage and δ = 0.4 (α = 0.05) the probabilities to reject H_0 _after the first stage are Pr(*p*_1 _≤ α_1_) = 0.496 and Pr(*p*_1 _≤ ) = 0.461, respectively. The probability to stop without rejecting H_0 _is Pr(*p*_1 _≥ α_0_) = 0.023 irrespective of truncation. With the fixed sample size of 100 per stage the expected total sample size is 200 - 100·Pr(stop after first stage). This expected total sample size is 148 for τ = 1, but 152 in case of truncation. Hence, the slight increase in power is connected with a larger expected total sample size.

An a priori fixed sample size for stage two is uncommon within an adaptive design. Instead, a sample size reassessment can be carried out during the interim analysis [11]. Using *p*_1 _and the difference and variability observed in stage one, we simulated the sample size for stage two needed for an overall power of 80%. The results (not shown) indicate that, in this case, the application of the truncated product method can lead to a smaller expected total sample size.

Nevertheless, there is still a smaller probability to stop the trial after the first stage when the truncation is applied. That is a clear disadvantage in clinical development where early decisions are desirable. Therefore, despite the (small) improvement in terms of power, a truncation does not seem to be preferable within a two-stage adaptive design when α_0 _< 1.

### The case α_0 _= 1

As mentioned in the introduction, α_0 _= 1 can be a suitable choice, for example when establishing a dose-response relationship. The choice α_0 _= 1 leads to the same rejection boundary *c*_α _for the interim and the final analysis, respectively. Hence, there is α_1 _= *c*_α _and . Since *c*_α _<, the expected total sample size is decreased due to truncation even in case of a fixed sample size for stage two. For instance, in the case of 50 observations per group and stage and δ = 0.4 (α = 0.05) the probability to reject H_0 _after the first stage is Pr(*p*_1 _≤ α_1_) = 0.342 for τ = 1, but Pr(*p*_1 _≤ ) = 0.354 for τ = 0.5. The resultant expected total sample sizes are 166 and 165, respectively. Therefore, a gain in power would be of more importance in case α_0 _= 1.

**Figure 2:**
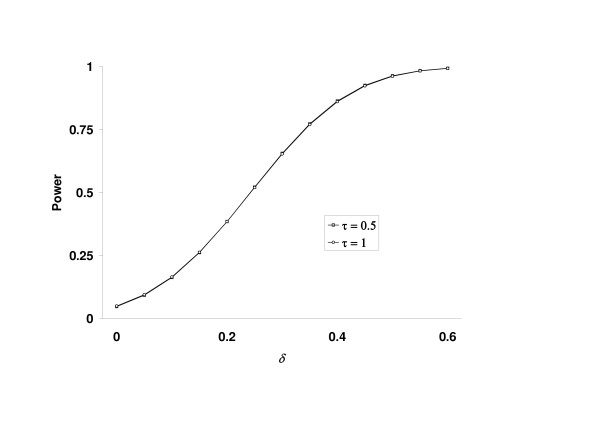
Power to reject H_0 _in a two-stage design with α_0 _= 1. (50 observations per group and stage, combination of *t *tests, one-sided, α = 0.05)

However, as demonstrated in Figure 2 there is hardly any difference in power between the choices τ = 0.5 and τ = 1. Nevertheless, the application of the truncated product method is preferable in the case α_0 _= 1 because there is a lower expected total sample size and a higher probability to reject H_0 _already after the first stage.

The value  increases with a decreasing truncation point τ. Hence, in order to increase the probability to reject H_0 _after stage one, one may argue that a smaller value of τ is preferable. However, this is not the case because the overall power depends on the choice of τ, too. For example, consider 50 observations per group and stage and δ = 0.4 (α = 0.05) again. In this case, the overall power is 0.861 for τ = 1, 0.864 for τ = 0.5, but only 0.830 for τ = 0.2.

We now present results for adaptive designs with three and four stages, respectively, and α_0 _= 1. Again, the behaviour of the strategies is investigated for fixed sample sizes in the separate study stages without including the option for sample size reassessment. The trial can be terminated with the rejection of H_0 _after the *j*-th stage if  in case of τ = 1 or if  in case of truncation. For up to four stages, Table II displays the boundaries *c*_α _and  for τ = 0.5.

The choices τ = 1 and τ = 0.5 were compared in a Monte Carlo simulation study performed using SAS version 8.2. For each configuration, 10,000 simulation runs were created. Table III shows the overall power and the expected total sample sizes. Always, the truncation is more powerful than the choice τ = 1, however, the difference in power is small. Furthermore, as in the case of *k *= 2, the expected total sample size is smaller when the truncated product method is applied (α_0 _= 1). The decrease of the expected total sample size is more pronounced for larger values of *k*. Therefore, the truncation can be recommended again. It reduces the expected total sample size without a loss in power.

**Table 3:** Simulated power to reject H_0 _and expected total sample sizes in three- and four-stage designs with α_0_ = 1 (50 observations per group and stage, combination of *t *tests, one-sided, α = 0.05)

	δ =	0.1	0.2	0.3	0.4	0.5
3 stages						
Overall power	τ = 1	0.198	0.498	0.789	0.950	0.993
	τ = 0.5	0.198	0.502	0.799	0.953	0.993
Expected total sample	τ = 1	293.3	278.7	250.0	213.7	179.6
size	τ = 0.5	292.7	276.9	247.1	209.9	176.1

4 stages						
Overall power	τ = 1	0.230	0.590	0.883	0.984	0.999
	τ = 0.5	0.233	0.596	0.888	0.985	0.999
Expected total sample	τ = 1	389.0	360.0	308.5	254.1	207.6
size	τ = 0.5	387.6	356.2	302.5	246.8	202.3

## Discussion

In this section we only consider the case α = 0.025 and α_0 _= 1. The first example discussed in this section was presented by Bauer and Röhmel [6]. In a two-stage dose-response study the effect of a new drug on blood pressure was investigated. Assume that the trial would have started with two medium doses. The *p*-value for the one-sided *t *test between these two doses in the interim analysis was *p*_1 _= 0.206. Thus, the study continued with the comparison placebo vs. a higher dose, and the second stage led to *p*_2 _= 0.0178. The product in the final analysis was p_1_p_2 _= 0.00367, the corresponding overall *p*-value of the non-truncated product test is 0.024. Hence, the combination test is significant even at the 0.025 level.

**Figure 3:**
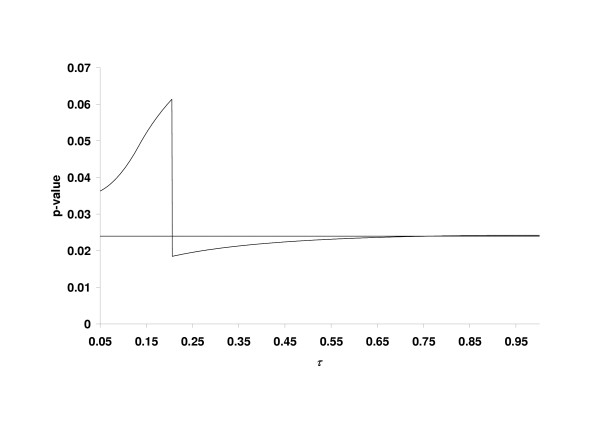
The overall *p*-value of the final analysis based on the combination of *p*_1 _= 0.206 and *p*_2 _= 0.0178 (first example) in dependence of the truncation point τ. The horizontal reference line corresponds to Fisher's product criterion.

Figure 3 shows the overall *p*-value of the combination test in case of truncation. Note that TPM *p*-values may be calculated using a C++ code offered by Zaykin et al. [7] which is available at , in addition, the method is implemented in the SAS procedure psmooth. There is no large influence of τ as long as this truncation point is larger than max(*p*_1_,*p*_2_). When τ is slightly smaller than max(*p*_1_,*p*_2_), i.e. for τ → max(*p*_1_,*p*_2_) with τ < max(*p*_1_,*p*_2_), the *p*-value reaches a local maximum of 0.061. For τ < min(*p*_1_,*p*_2_) the *p*-value equals 1. Hence, a too small choice of τ is risky. Thus, the analysis of this example may be a further indication that the choice τ = 0.5 is reasonable. In fact, in this example any τ > 0.206 would have been a powerful alternative to Fisher's criterion.

The second example is a hypothetical clinical study with two stages. We consider a scenario as Bauer and Köhne [[4], p. 1038] in their example. A clinical trial investigates a new therapy for an indication in which no efficient standard therapy is available. For the first stage five individual endpoints have been selected. The first stage's sample size is 30 each in the therapy and the control group. The changes to the baseline measurements of the five endpoints were combined into a single generalized least squares (GLS) criterion according to O'Brien [12], and the first stage's *p*-value was *p*_1 _= 0.1758. Hence, the study continued.

For the second stage the set of five endpoints may be reduced for different reasons such as observed effects and variability, burden to the patients, and costs. The test statistic for the second stage was again the corresponding GLS criterion. In this example, this led to a *p*-value of a similar magnitude as in the first stage: *p*_2 _= 0.1517. Therefore, in the final analysis we have *p*_1_*p*_2 _= 0.0267, and the corresponding overall *p*-value is 0.1233 when Fisher's original product test is applied. The overall *p*-values for different choices of τ are displayed in Table IV. Here, the TPM gives a smaller overall *p*-value than Fisher's method for all considered values of the truncation point with the exception of τ = 0.1. However, that value is smaller than min(*p*_1_, *p*_2_). In this example α_0 _= 1 may be appropriate because no efficient standard therapy is available, the sample size of stage 1 is relatively small, and there might be only one endpoint showing a difference between the therapy and the control group.

**Table 4:** The overall *p*-value of the final analysis based on the combination of *p*_1_ = 0.1758 and *p*_2 _= 0.1517 (second example) in dependence of the truncation point τ, τ = 1 corresponds to Fisher's product criterion.

τ	*p*-value for TPM
0.1	1
0.2	0.0801
0.3	0.0964
0.4	0.1064
0.5	0.1130
0.6	0.1174
0.7	0.1203
0.8	0.1221
0.9	0.1230
1.0	0.1233

## Conclusion

The application of the truncated product method instead of Fisher's combination test within an adaptive design hardly changes the overall power. Therefore, to decide whether or not a truncation is useful one should focus on the probability to stop early and on the expected total sample size. According to these criteria, a truncation seems to be preferable in case of α_0 _= 1, but not for α_0 _< 1.

A variety of other combination functions exists [13], for example, the inverse normal method was proposed for adaptive designs [14]. According to Rice [15] Fisher's test is "inappropriate when asking whether a set of tests, on balance, supports or refutes a common null hypothesis ... because ... Fisher's statistic is more sensitive to smaller, as compared to larger, *P*-values" [[15], p. 303–305]. In contrast, the inverse normal method is not differentially sensitive to data that support or refute a common null hypothesis. Thus, one may argue that the inverse normal method is more appropriate for an adaptive design if each stage tests the same null hypothesis. However, in the context of a dose-response study, discussed here as a motivation for α_0 _= 1, different doses may be tested in different stages, that is, the hypotheses tested change. The resultant question is whether at least one stage is significant, and a high sensitivity to small *p*-values is desirable. Consequently, Fisher's test or TPM are appropriate. An additional advantage of these two combination methods is that an early termination with rejection of the null hypothesis is possible with α_0 _= 1 and a full level α combination test at the end.

There is also some literature related to the efficiency of adaptive designs, and to the choice of combination functions. Wassmer [16], for example, compared Fisher's product criterion with an alternative adaptive design proposed by Proschan and Hunsberger [17] based on a conditional power function. Wassmer [16] concluded that "no substantial differences between the procedures were found in terms of rejection regions, power, and expected sample sizes". One of the first to investigate optimal adaptive designs for the control of conditional power were Brannath and Bauer [18]. They constructed two-stage designs with overall and conditional power, which minimize the expected sample size for different specifications of the alternative. It transpires that there is a variety of different options to combine *P*-values and there is no consensus on the best method to use. In this paper we improve under special conditions Fisher's combination test using the truncated product method.

It is worthwhile to note that the truncation point τ must be specified a priori in the study protocol. Unless determined a priori, the truncated product method can be misused to alleviate an observed significance. A post-hoc choice based on the observed maximum of the individual *p*-values is therefore not permitted. As discussed above, τ = 0.5 may be a suitable choice. A further argument for this choice is that those *p*-values are excluded from the product that indicate a difference in the unanticipated direction. Note that the truncated product does not follow a χ^2^-distribution. Thus, a penalty results for the exclusion of large *p*-values. Nevertheless, this exclusion can be advantageous as demonstrated by Zaykin et al. [7] and above for the case of adaptive designs.

For the presentation of the power a one-sided significance level of α = 5% was chosen in this paper. However, completely analogous results can be found in case of α = 2.5%. Regarding the choice of α for one-sided tests it is referred to Neuhäuser [19].

## Appendix

The power of a two-stage test according to Bauer and Köhne [4], that is, a combination with τ = 1, is given e.g. by Wassmer [[20], p. 833].

In case of truncation with τ = α_0 _> α the power is



where *f*_δ _denotes the respective density under the alternative δ [20]. In case of truncation with τ > α, but α_0 _= 1, the power is



Wassmer [20] presented a SAS/IML program to calculate the power for the two-stage test without truncation. Modifications of this program were used to calculate the different powers given above.

## Abbreviations

TPM – truncated product method

## Authors' contributions

MN performed most of the statistical analyses and drafted the manuscript. FB participated in the statistical analyses and helped to draft the manuscript. Both authors read and approved the final manuscript.

## Pre-publication history

The pre-publication history for this paper can be accessed here:


